# Dr. Stubb's Visit to the Parisian Lunatic Asylums

**Published:** 1848-01-01

**Authors:** Henry Hunt Stubb


### Original Communications.

### NOTES ON THE PARISIAN LUNATIC ASYLUMS.

BY HENRY HUNT STUBB, M.D.

*Saint John's, Newfoundland, August* 1847.

Having recently visited the asylums for the insane in Paris and its neighbourhood, it may not be uninteresting to the profession if I venture to communicate the observations I have made.

Sent purposely from this country, I first visited, in May and June last, several of our own asylums, especially those of Hanwell and Bethlehem,—the former under the guidance of Dr. Conolly; and, besides my particular object in afterwards examining the French asylums, I was so much struck by the happy state of the insane poor at Hanwell, that I felt desirous of comparing with it the state of the same class of beings at Paris,—the city which first saw these afflicted visited by humanity, in the person of Pinel.

By permission of the French authorities, I visited Charenton. By M. Battelle, director of the hospices and hospitals of Paris, I was conducted through the asylums of La Salpêtrière and Bicêtre, and the farm of St. Anne

My first visits were paid to La Salpêtrière, an institution containing 5000 females, of whom, on an average, from 1400 to 1700 are insane poor of all ages (formerly of old age only). Attached to the asylum are three resident physicians, M. Lelut being the principal, and two physicians-in-chief, non-resident, MM. Falret and Mitivié.

I was taken to every part of this colossal establishment by M. Battelle, who, with an earnestness that could spring only from a heartfelt interest in the subject, explained minutely the actual state of everything connected with the department for the insane, as well as the successive steps by which the whole had been brought to its present state of perfection,—a designation which I think is justly merited. For if much remains to be done, or is rather being done, in exchanging old things for new, in parts of this princely pile of the days of Louis Quatorze; there is, nevertheless, at the present moment an ample provision of means—perhaps superior to that of any other asylum in the world—for the curative treatment of the recently insane, and for the care and comfort of those afflicted with chronic insanity. And it is somewhat worthy of mention, that the saloons of the indigent insane are adorned with marble, polished oak, and spotless white draperies, yet it need not excite a word of cavil, for, as my excellent conductor observed, solid oak and marble would last for centuries, and such asylums were not built for a day.

With regard to the inmates of this institution, as of Hanwell, it may be said, such happy influences surround them, that their insanity is veiled from the passing observer; and when amongst them, the question arises involuntarily in the mind, Are these really insane? In La Salpêtrière the insane are placed in five distinct divisions of the buildings, which are for the most part new, and, under the direction of M. Battelle, so arranged as to present a succession of apartments equally remarkable for size and elegance, and admirably adapted to the comfort of the patients. For the sake of classification the divisions are appropriated as follows :—One for insane epileptics, two for the chronically insane, and two for the insane under treatment, or the curable. For those under temporary excitement, there is enclosed a large grassy court, with Swiss cottages disposed around, containing one room each. To each division is attached an extensive airing court, with trees, small gardens, and grass-plots, for exercise and amusement. For the use of the whole establishment, there are large kitchen-gardens within the walls, which are cultivated by the insane of this asylum, assisted by others from the Bicêtre. Within the buildings are found large dormitories, of superlative cleanliness, well lighted and ventilated, the floors of oak, beautifully waxed. The beds are arranged on a raised platform on either side, leaving a wide passage through the centre of the ward ; the bedding is remarkable for snow-white linen, heavy blankets, and numerous thick mattresses. The windows of the dormitories are large, and open in six compartments ; they have curtains, and are unguarded by iron-sash, bar, or wirework, excepting in certain wards having an exterior aspect. It is said that the window-panes are never broken by the patients, and the small high window is never introduced here.

The iron bedstead for a patient in a fit of epilepsy is worthy of notice. The upper half turns down, to admit of easily placing the patient in bed ; and the head, foot, and half of the sides are padded, to prevent injury during a fit.

For the dirty a mass of loose straw,* with a sheet stretched firmly round it, is preferred for bedding, there being a hole in the centre of the bed conducting to a copper pan in a drawer beneath.

The dormitories open by glass doors into convenient day-rooms, where I found the patients at various times, busily occupied with the needle, chatting and singing.

The general arrangements are so intended, as to divest the minds of the inmates of any feeling whatever of imprisonment. And whether occupied within doors, or enjoying the fresh air without, the insane at La Salpêtrière appeared free and happy. The diet at this establishment is unexceptionable. The dining-tables are covered with oil-cloth. The dishes and spoons are of metal, and the knives and forks of the ordinary kind.

The bath-rooms, in accordance with the general air of luxury, are very large and lofty, the walls of painted stucco, and the windows curtained. Each bath has a moveable cover, with a narrow opening for the neck, but too small for the head to pass through ; the practical use of which I observed in a patient who kicked violently and twisted about her body in the bath like an eel, but tried in vain to get her head down.

The clean linen saloon is one of the finest and handsomest apartments in La Salpêtrière, containing the general supply for the hospices and hospitals of Paris,—all sewn, washed, and assorted by the insane.

* At Charenton and Bicêtre, sea-weed is substituted for straw.

Shortly after my arrival, I was present at one of M. Falret's weekly reunions of the insane, when recitations and singing took place by the patients, who appeared much interested, especially in the songs, when many united their voices. They sat, in number about a hundred, variously dressed, and sewing, except when required to recite. Their cheerful countenances when singing showed the pleasure they experienced, and the general occupation combined with amusement—the physician and attendants being present as encouraging friends,—could not but have a beneficial effect upon their disordered minds, whether in promoting the dawning convalescence of some, or in soothing the morbid irritability of others—a beneficial, curative effect.

With regard to the quietness of so many assembled lunatics, in different stages of various mental maladies, M. Falret observed, that he had at length succeeded in obtaining complete control over the actions of his patients, (however difficult it might be to direct their thoughts in any given channel !) Now, as no effort was observable on the part of the physician or attendants in maintaining quietness and decorum, the influence of their presence alone was evidently sufficient to induce the patients to control themselves,—a great step as it appears to me.

Amongst the hundreds of insane in this asylum, in vain did I look for the furious maniac ; almost in vain for the excited,—with the exception of the female in the bath, above mentioned, and two whom I met walking about the grounds in strait-waistcoats. 'Tis true, I encountered an old woman who had been an inmate for thirty years, who talked herself into a violent rage ; and, whilst in one part of the garden, I heard an outcry from a patient who had become suddenly excited, in a neighbouring part of the grounds ; but the ordinary calmness of the numerous patients, consequent upon their excellent general treatment, is the reality which impressed itself on my mind from my visits to La Salpêtrière.

Besides those whom I saw in the day-rooms, dormitories, and airing-grounds, I observed some hundreds of insane employed in various ways, —spinning, sewing, tailoring, washing, hanging out clothes to dry, gardening, and farming ; all quiet and orderly, under the superintendence of respectable-looking attendants.

This asylum is heated by means of circular stoves, which are placed, guarded, in the centre of the saloons and dormitories. But an apparatus for heating the buildings by steam is about to be introduced by M. Battelle, on the plan observed by that gentleman in England ; which, in so large an establishment as La Salpêtrière, will prove as economical as effective.

The chapel, on an equally grand scale with the other buildings, can contain as many as Notre Dame—viz., 2000 people ; it is octagonal, and divided into so many compartments, some of which are set apart for the insane, who frequent this place of worship with the best effect upon their minds, and always behave with reverence and decorum. I entered it in the evening, and observed about a hundred people there, some praying aloud. To the insane, the benefit of such attendance, and of the religious instruction carefully and wisely administered under the guidance of the Abbé Christophe, is constantly extended.

On quitting La Salpêtrière, I could not refrain from observing to M.

Battelle, that the realization of such perfect cleanliness and elegance, not to say magnificence, in a public charity, was extremely difficult of belief, even to an eye-witness, and the fact might be fairly doubted by others; whilst the knowledge of this being the asylum provided for the poor and miserable, the epileptic and insane, savoured more of romance than reality. But it is indeed a reality, worthy of a great nation; and a striking example of the true practical genius of Christianity!

It is scarcely necessary to add, that there is an infirmary and apothecaries' establishment in La Salpêtrière.

I have described the beneficial effect produced on the insane by M. Falret's reunions for singing, recitation, &c. At the Bicêtre I was present at a more remarkable reunion—a reunion of idiots! These unfortunates had been taught dancing and singing, fencing, military drill, recitation, writing, and arithmetic, with such Herculean pains of philanthropic hearts as few can imagine!

The previous day I had visited the school in which these lessons were received in a dull, obtuse, mechanical manner by the poor pupils, who were drawling in the monotonous tones of a village school, and it was only on observing the revolting countenances around, that a visitor became necessarily aware that he was amongst idiots. What an occupation—the cultivating of minds all but utterly sterile! And what can repay the originators of this divine work? Unless it be the consciousness that they, and the labourers in it, are permitted to clothe with sense and reason, however limited, those whom a mysterious Providence has placed on the earth clothed with something like brutality!

The holy attempt to awaken faculties hitherto dormant, to restore to themselves and to God, as it were, these lost minds, demands the praise of mankind, as the most stupendous of human endeavours,—endeavours happily not without success; gleams of intelligence shine occasionally through the mental darkness, and these, fostered during days, months, and years, have, in certain instances, increased in number, and have even become combined into a steady, continuous, mild light of reason, shining from the erewhile vacant eyes of the idiot—Charles Emile, for example! And this is the recompence of the teacher!

At this reunion eighty-four boys, idiots and epileptics, were present, and went through their various exercises with considerable skill and great propriety, under the direction of M. Vallée, their superintendent and instructor. M. Battelle, who accompanied me, was much moved, and said he could scarcely refrain from shedding tears as often as he came amongst these children. My own feelings at witnessing, for the first time, such an unparalleled scene, were painful, as well as pleasing in a high degree.

The song of these idiots is worthy of recollection, and I take the liberty of inserting it, as sung by the children, whose destiny is its theme; it was peculiarly affecting.

> " Transformons le monde où nous sommes,
> Reveillons nos sens endormis,
> C'est le travail qui fait les hommes,
> Travaillons, travaillons, amis.

"La fleur a sa beauté prémière,
L'oiseau rend des sons differents,
Et le bon Dieu dans sa lumière
Sourit aux petits comme aux grands.

"Chacun a son lot d'héritage,
Chacun a des dons définis,
Sommes nous exclus du partage ?
Enfants que Dieu n'a pas benis !

"Non ! puisqu' ici l'on récommence,
Tous nos organes imparfaits,
Et qu'on féconde la sémence,
Des biens que le ciel nous a fait."

Two shocking looking idiots were seated during the exercises of the rest a little apart, their useless legs strongly bound up with leathern supports. These I thought must be indeed hopeless cases. Nothing could exceed the vacuity of their countenances, with large protruding lustreless eyes, and tongues lolling out of their mouths, nor the wretched appearance of their bodies, with paralytic arms and legs. I was therefore not a little surprised to see these two scarcely human objects brought in their chairs to a small table upon which dominoes were placed, with which they played a game; and it became evident that all was not lost to the mind even for them—they became interested and excited, and a hideous joy was expressed by the winner.

If, then, they had learned something, no matter what, and evinced interest in the lesson, the elements of mental advancement were present in them also.

How much is not due to the ardent M. Voisin, and others, who instituted this anomaly—a school for idiots!

From this school I had the pleasure of bringing and presenting to Dr. Conolly a crayon drawing, executed with great fidelity and skill by one of the most improved of the boys—a most interesting proof of what may be accomplished in such a school.

After the reunion, I went to the workshops, where these idiots were employed more or less usefully, and Charles Emile I observed using a jack plane with tolerable steadiness, grinning and smiling, quite pleased to be doing something; it may be, to be thought capable of doing anything. I may here observe, that the same satisfaction was expressed in his face, accompanied frequently by a short cry of exultation, when successful in the previous exercises, as he generally was, in naming, for example, the mathematical figures placed in his hand, the odours applied to his nose, and different substances to his tongue; showing the successful education of the senses, which seemed scarcely existing in him but a few years since. He was, moreover, greatly delighted when purposely told he was wrong by M. Battelle, at showing that he had rightly written and named certain arithmetical figures on a board. He had learned something correctly, knew it to be correct, and took pleasure in having learned it—no mean advancement from the former idiotic state, horrible to contemplate, of this individual, who is described as a voracious, cruel, filthy animal, with the worst of brutal propensities.*

* Il est idiot dans presque toutes les virtualités de son être, idiot dans ses sentiments, idiot dans ses facultés de perception, idiot dans ses pouvoirs intellectuels, et

It took six months of infinite pains to effect any change for the better in this being. His present state is the result of seven years' labours, as I was informed by M. Vallée.

That schools for the insane and idiotic may soon be instituted in our own asylums to rival those of MM. Falret and Voisin, is my earnest hope!

Comparing the state of the insane poor at Hanwell, La Salpêtrière, and Bicêtre, I find plenty of evidence of the enlightened care of which they are equally the subjects; but whilst there are many points of resemblance in the practice of the French and English physicians attached to these institutions, there is to be remarked one prominent difference between them—viz., the use of mechanical restraints in the one, and their entire disuse in the other. M. Falret placed in my hand his pamphlet on the establishment for the insane at Illenau, in which he speaks openly and determinedly in favour of those mechanical restraints, the very absence of which at Hanwell places that institution, in my opinion, in advance of the French asylums.

M. Falret says, "Quelque bonne opinion que nous ayons de nos confrères d'outre mer, et en particulier du Dr. Conolly, nous ne croyons pas qu'ils soient parvenus à rendre inutiles tous les moyens de restriction. Pour qui connait les aliénés, leur 'no-restraint' est une fiction, et une simple substitution d'un moyen à un autre: j'ajoute avec une profonde conviction que le 'solitary confinement' dans une chambre matelassée est une mode de répression mille fois plus pénible, plus restrictif de la liberté, que la camisole, et qu'il est contraire au premier précepte du traitement des aliénés agités, qui consiste à les placer dans les conditions les plus favorables aux exercises en pleine air, que la nature leur commande si impérieusement. Nous réconnaissons néanmoins avec plaisir que la réaction actuelle de quelques médecins Anglais contre les répressions peut-être d'une utilité générale, et qu'elle était indispensable dans un pays où l'on avait épuisé l'arsenal des moyens mécaniques pour torturer les aliénés, et où j'ai constaté encore en 1835, l'emploi des chaînes, même dans l'asile de Bedlam."

Without noticing the latter part of these observations, I have only to extract from the rest the plain opinion of a highly distinguished French Physician attached to La Salpêtrière, (and more or less assented to by his brethren,) that the so-called non-restraint is a fiction, consequent upon the excessive reaction of some of the English physicians, against the old system of restraint; that it is not what it pretends to be, but merely the substitution of one means for another; and, finally, that solitary confinement in a padded room is not only a mode of restraint a thousand times more painful and restrictive of liberty than the straitpar l'état de ses sens, dont les uns sont obtus et les autres trop irritables. Il se trouve encore sous ce rapport hors d'état de pouvoir convénablement s'harmonier avec le monde extérieur. Sous le rapport des penchants que nous partageons avec les espèces inférieurs, il n'y a de bien marqué chez cet enfant qu'un appetit vorace et glouton, qu'un érotisme hideux, et qu'un instinct aveugle et terrible de destruction: l'animal se montre chez lui tout entier. Toutes les facultés de perception sont à l'état rudimentaire: on a une peine incroyable à le faire sortir de son individualité; on ne serait pas bien loin de la vérité en disant que pour cet enfant la nature est presque complètement voilée.—From the Résumé of his case, by M. Voisin.

waistcoat, but that it is contrary to the first principle of treatment regarding excited lunatics.

I should have thought the personal knowledge which M. Falret has of Dr. Conolly, and the respect which I have heard him express for him, would have prevented his making such an unlimited assertion as the last—knowing also, as he does, that Dr. Conolly, with all his great experience and eminently mature judgment, is the earnest advocate and promoter of that system which M. Falret appears so much to disdain, and to which, it is but justice to add, that neither he nor any other physician of the Parisian asylums has given a practical trial.

To this opinion of M. Falret, may be best opposed that of our first of English physicians regarding insanity, supported by the valuable non-professional testimony of the Middlesex magistrates—visitors of Hanwell. We find both in the report of that asylum of December 31st, 1846. Dr. Conolly writes, "On the 21st day of September last, seven years were completed, during which no strait-waistcoat, muff, leg-locks, hand-cuff, coercion chair, or other means of mechanical restraint, have been resorted to in the Hanwell Asylum, by night or by day. In these seven years, 1100 cases have been admitted and treated entirely on the non-restraint system, and the number of patients in the asylum has during a great part of the said period amounted to nearly 1000. There are still some asylums in England, Scotland, and Ireland in which such means of restraint are employed and defended; and travellers from various parts of the Continent, and from the United States of America, apparently prepossessed in favour of such ancient and forcible methods of control, continue to pay hasty visits to Hanwell, and to publish opinions condemnatory of the non-restraint system. In the annual reports of past years, when the experiment was but in an early stage of its progress, and when it was embarrassed by many difficulties, I refrained from engaging in any controversy on the subject, being satisfied that the result would furnish the best test of its being rational and judicious, as well as humane. If such results had not appeared, it would have been my duty to alter or relinquish any other particular in the treatment of the patients. Now, after seven years' patient trial, during which the non-restraint system has been introduced into many other asylums without the occurrence of any accident against which mechanical restraint would have afforded security, I do not think it desirable more particularly to notice the opinions of writers, who have sometimes appeared to visit Hanwell more prepared to argue than to observe; nor should I deem it necessary to refer to this part of the treatment if it were not that I consider it still requisite to remind those who are most anxious to adopt it, that certain conditions are essential to its being successfully maintained."

The Committee of Visitors of Hanwell, in their report of January 19th, 1847, say:—"It affords them the utmost satisfaction, after the experience of another year, to be enabled to speak in terms of still higher praise of the humane system, which has happily been progressing in the establishment for some years past, and they have not had the slightest cause to regret the abolition of all restraint, which they attribute to the zealous and efficient manner in which the duties of the officers and servants have been performed. The committee are anxious to draw the attention of the court to the report of the visiting physician now presented, as containing matter of great interest and importance, on the humane and successful management of the insane without restraint in public asylums. The subject is so ably and convincingly brought forward by Dr. Conolly, that the committee do not think it necessary to add more than their entire approbation and concurrence in the sentiments so well expressed by him.

"Signed CHAS. AUGUSTUS TULK, Chairman."

Dr. Falret and other advocates of mechanical restraints may find the opposite system thoroughly explained in the lectures of Dr. Conolly, published in the "Lancet," in 1845, and in his work "On the Construction of Lunatic Asylums," &c., and convince themselves that non-restraint is no fiction, but common-sense reality, practised in several large asylums on this side of the water with the best effects.

An important amelioration in the care of lunatics, which must eventually be adopted in every asylum, both European and American, despite the opposition of those who condemn it without trial. An amelioration, however, which emanating from Lincoln and Hanwell, will not, perhaps, be universally adopted, until the natural *amour-propre* of physicians in asylums shall cede to their love of humanity, and the interests of medical science; and this may be last expected of the French, from their national character—in physicians, from preference for the results of their own labours in the case of insanity; and in the patients, from that vivacity and great nervous irritability, which falsely seem to demand mechanical restraints, when developed in a high degree by the invasion of insanity. Now, although at La Salpêtrière, during my visits, I observed strait-waistcoats on two patients only, yet it is not denied that these and other mechanical restraints are constantly used; suicidal patients, for example, are tied to the beds they lie upon. And at Bicêtre, where the physicians, M. Voisin and others, are men of equally high distinction with those of Salpêtrière, I observed the bad practical effect of mechanical restraints. Thus, five men were lying bound in bed with strait-waistcoats on and bandages over their eyes, in the ward for the Agités; and in the airing grounds of this class of patients were six men in strait waistcoats, and several fastened in chairs—those in bed writhed and growled savagely, unquestionably in a great degree from the irritation produced by these restraints. May I be permitted in this place to endeavour to show why the true meaning of the system of non-restraint, which is practised in England, is so grievously misunderstood by the French physicians.

I will premise that a madman, from his inability to govern his thoughts and actions, is placed, by the common consent of mankind, under the restraint of a physician, who is entrusted with moral and physical power over him.

The physician formerly used mechanical means of restraint as a matter of course, the necessity for such things never having been questioned. In the present day, however, the necessity for restraining the violent motions of a madman, by the application of mechanical means of restraint to his person, is not only questioned, but explicitly denied.

The first step towards the removal of this great error in medical practice—viz., the abolition of the more degrading mechanical restraints —we identify with the emancipation of the insane from chains, fetters, and dungeons, by Pinel, who, by a spontaneous act of the highest charity, amidst the temporary horrors of the French revolution, rid the world of horrors hitherto permanent. But the abolition of the less infamous, but equally unnecessary, mechanical restraints, has been the recent work of English physicians; of Dr. Charleston and Mr. Hill, of Lincoln, and of Dr. Conolly, of Hanwell, who (if Pinel destroyed the illusion that the insane are necessarily ferocious, therefore to be chained) destroyed the remaining illusion that excited lunatics are necessarily dangerous, therefore to be manacled. This disuse of every mechanical restraint whatever, in the treatment of the insane, by the English physicians above-named, and their followers, has been somewhat erroneously called the system of non-restraint, which M. Falret calls a fiction, because restraints external to the persons are really made use of, as in the padded room; but non-*mechanical*-restraint is no fiction.

If M. Falret and his brethren say it is against that first principle of treatment, exercise in the open air, to place a lunatic, when in a paroxysm of excitement, for a short time in a room where his limbs have complete freedom of motion, the said principle being sufficiently put in practice at other times, I am at a loss to comprehend their meaning; and I must observe, that to term temporary seclusion solitary confinement, is erroneous, because it leads to the false conclusion that the patient is subjected to a confinement of indefinite duration and painful character.

Now, the principle of enjoying the open air in a strait-waistcoat, excited from within and bound up without, appears to my understanding to be contrary to something like a first principle—viz., the removal of all causes of irritability from a patient suffering under intense nervous excitement. A principle which originated the padded room, on two grounds—the necessary absence of mechanical restraints, and of all objects which, when presented to the external senses, excite the mind within. The decision on the point at issue, however, must rest on this, *cæteris paribus*, the comparative facility of recovery of excited lunatics from their paroxysms under the systems of mechanical restraint and non-mechanical restraint; and the comparative humanity of the two systems. If it can be shown that the cases are in favour of restraint, non-mechanical restraint must fall to the ground; but in all candour, let not the question be assumed, by calling the latter system a fiction. In my remarks upon the system of restraint, I have particularly alluded to the opinions of M. Falret, but it should be understood that mechanical restraints are used by Drs. Foville, Voisin, and the other physicians of Charenton, Bicêtre, and La Salpetrière, and I have reason to believe they are used in the asylums throughout France.

Thus, in the "Annales d'Hygiène Publique" of July, 1847, p. 48, Dr. Brierre de Boismont alludes to the system of non-restraint practised in Hanwell, which asylum he had hastily visited. He attributes the success of the system to the incurable state of the majority of the inmates, and he says, "Nearly all the individuals who come into this house have been treated elsewhere, and are consequently in a very favourable state for the non-restraint system." This is rather poor argument, and I would refer M. Brierre de Boismont to the Bethlehem reports of 1844, 1845; Bethlehem admitting none but recent cases of insanity. In the report for 1844, p. 49, we read:—" From the peculiar character of the cases received into this hospital, it is deemed inexpedient rather than impracticable to adopt the principle of dispensing wholly with restraint, under all circumstances, yet every opportunity is taken of confining it within the narrowest limits." Thus, it is found to be practicable in recent cases, if thought necessary, as it is by the advocates of non-mechanical restraint; and this evidence from the physicians of Bethlehem plainly shows that the use of mechanical restraint is a matter of choice, in recent as well as chronic cases of insanity. The fact that recent cases are more likely to give trouble,—that is, to require more restraint in periods of excitement,—than chronic cases, and that such periods are more constant, proves nothing in favour of mechanical restraints.

In the report above mentioned, we find also that mechanical restraints are nearly abolished; and to this I may add my own observations in 1847, when I saw no instance of mechanical restraint:

" Average weekly number of patients in restraint at Bethlehem in—

| | | | |
|---|---|---|---|
| 1839 | $11\frac{33}{52}$ | 1843 | $3\frac{4}{52}$ |
| 1840 | $13\frac{39}{52}$ | 1844 | $1\frac{35}{52}$ |
| 1841 | 9 | 1845 | $1\frac{20}{52}$ |
| 1842 | 3 | | |

" The experience of Bethlehem, however, shows that in any asylum *mechanical restraints both irritate and humiliate* a patient," &c.

P. 50. " Nearly forty-one per cent. of the curable patients admitted this year were classed as violent or dangerous, above fifty per cent. of the total admissions thus consisting of a class which, a *few years ago, would, as a matter of ordinary precaution and humanity, have been subjected to rigorous personal restraint. Experience has, however, shown, and every year confirms, not only the wisdom of a totally different system, but that restraint is a highly exciting cause of suicide;* and the fact that no untoward circumstance has occurred in Bethlehem, with so large a number of dangerous patients, while mechanical restraint has not been used for two patients a week, is a most striking illustration of the advantage of this system." At the present moment the padded room is used at Bethlehem, and the practice of the physicians seems to have nearly passed through the transition state between the use and disuse of mechanical restraints. A proof of the superior practical benefit of the latter system in recent cases, when it obtains a fair trial. And a sufficient refutation of M. Brierre de Boismont's opinion concerning the non-mechanical restraint system in acute and chronic insanity.

In conclusion, I would ask M. Falret, whether the seclusion of the Swiss Cottage, for agités, is not tantamount in principle to the seclusion of the padded room?

The famous Hospice of Bicêtre, besides its 2000 infirm old men, contains 850 lunatics. There are nominally two resident physicians, but only one is really resident. The patients are not so well classified as at La Salpêtrière, the wards containing each insane of all kinds. To both asylums, lunatics are sent, at any moment, by the Prefect of Police.

There are numerous single cells to be seen at the Bicêtre, very much on the old principle, strongly guarded by ironwork. For insane criminals, a building has just been completed, of a circular form, heavily barred with iron within, and surrounded by high walls without. It has eight divisions for six patients each, radiating from a central circular apartment, which has windows looking towards each division, to place the inmates under the constant observation of the keeper.

In connexion with the Bicêtre, although at some distance from it, is the farm of St. Anne, containing 150 acres of land, cultivated with the spade, by the insane; and bleaching grounds, where 4000 pieces of linen of 60 decimetres in length are bleached annually, also 20,000 couvertures. The insane whom I saw here employed were working machinery by capstans, for raising water. At this time there were 150 at work; sometimes so many as 500 insane are employed on this farm.

During my stay at Paris, I was invited to see the private asylum of MM. Falret and Voisin, situate a league and a half from the city, in a large park, beautifully embellished with trees, and diversified by lawns, woods, acclivities, flower gardens, fountains, a fish-pond, &c.; tastefully laid out, and presenting cottages and houses, in different parts, carefully secluded, and offering charming retreats for the higher classes whose mental weakness or disease demands such places of repose and retirement: whilst within the grounds there is also a variety of objects to please the eye, and to solicit bodily occupation,—gardens to cultivate,—poultry to feed,—pet birds and animals to be cared for,—retired walks, and pleasing prospects. I would add, kind friends in the proprietors and their families; and well-selected attendants, who, whilst always at hand, never obtrude themselves unnecessarily on the inmates. There remains, then, nothing to distress the patients, or remind them of their maladies; but everything is present to lead the mind from preying upon itself, to contemplate external things, and happily to recover a sound state of existence.

It would be strange if the aliéné did not encounter in this beautiful spot some well-known object to please and to remind of other days, either in the grounds, the gardens, or the farm-yard. And I cannot conceive a more fortunate circumstance for a lunatic of superior class, understanding, or habits, than to become an inmate of this abode. Whilst a parallel piece of good fortune for a patient of humble rank, is to have such a place as Hanwell or La Salpêtrière allotted for a home.

This establishment is so extensive, that the family of an insane person may, if they desire it, remain within the walls, with their domestics. I had the pleasure of dining in the garden with the two physicians and their families, and some of the insane. And I feel myself equally a debtor for the hospitality and kindness I experienced at the hands of Doctors Falret and Voisin, as for the opportunity of observing the practical working of the institution; whether at table with some of the patients, or in the grounds occasionally in sight of groups of others. At the time of my visit there were sixty inmates.

The royal asylum of Charenton is under the immediate authority of the Minister of the Interior, and contains in general from 485 to 500 insane. It is situated about two miles from the barrier, on the side of a hill, commanding a beautiful prospect of the Seine and Marne and surrounding country. The buildings are very extensive, and, from the steepness of the site, rise in terraces, which adds much to their effect, but detracts from their convenience, for the very precipitous and long flights of stone steps are neither commodious nor safe. The asylum is being entirely rebuilt, and the division for male patients is already completed. The general plan is for two-story buildings, with open corridors, forming three sides of squares in succession, which inclose the airing courts, and are open to the valley of the rivers.

The inmates are maintained at their own expense, regulated by the government as follows:—For the first class, 1300 francs; the second class, 1000 francs; the third class, 720 francs per annum. The accommodations are in accordance with the sums paid: thus, for the first class, a well furnished apartment is provided for each person, and an adjoining room for a servant; whereas patients of the third class sleep in common dormitories.

There is an elegantly furnished salon for the soirées, which frequently occur, when selections are made from the superior class of patients of both sexes. There are also an ordinary drawing-room, a billiard-room, library, &c.

The physician in chief is M. Foville, who is assisted by two resident physicians. I was sorry to see, that under them, also, the system of mechanical restraint was in full operation. Strait-waistcoats, coercion-chairs, bed-straps, &c. One man, strapped to a chair, had several cicatrices on his neck from suicidal attempts, which leads me to speak of the great tendency to suicidal acts prevalent in the French asylums. In this institution, for example, three weeks before my visit, a patient escaped from the female division of the house, and precipitated herself from the front of the chapel upon the stone pavement, sixty feet beneath. A week previous to this shocking event, a man hung himself, who had never, during a sojourn of ten years, shown any propensity to suicide. Another case shows that sleeping in a common dormitory does not prevent the act of suicide, when the suicidal determination is strong,—a patient having hung himself in one of these wards with such precaution that it was not discovered for some hours. The curtains of the bed hid this man from observation, and they have been discontinued, in consequence, in all the wards.

This tendency to suicide is in a great measure attributed by the English physicians to the use of mechanical restraints. Should not this cause the French physicians to pause a little, and consider well whether it be so or not?

I cannot conclude this letter without expressing my sense of the very great courtesy I experienced from all those to whom I was introduced, connected with the Parisian lunatic asylums; and I am convinced that I am no solitary example of an English member of the profession being well—nay, cordially received by our brethren in Paris.

The director of the Hospices and Hospitals, M. Battelle, evidently makes it a point of honour to spare no pains in assisting the researches of those introduced to him.

